# Improving Ram Semen Low Cryotolerance by Replacing the Seminal Plasma With That of High‐Cryotolerant Rams or Extender

**DOI:** 10.1002/vms3.70381

**Published:** 2025-04-28

**Authors:** Cemal Dayanıklı, Bülent Bülbül, Şükrü Doğan, Ebru Şengül, Mesut Kırbaş, Yavuz Kal, Mehmet Bozkurt Ataman

**Affiliations:** ^1^ Department of Breeding Techniques Sheep Breeding Research Institute Balıkesir Türkiye; ^2^ Department of Reproduction and Artificial Insemination Faculty of Veterinary Medicine Dokuz Eylül University İzmir Türkiye; ^3^ Department of Animal Breeding Bahri Dağdaş International Agricultural Research Institute Konya Türkiye; ^4^ Department of Reproduction and Artificial Insemination Faculty of Veterinary Medicine Selçuk University Konya Türkiye

**Keywords:** cryotolerance, extender, ram, semen, seminal plasma

## Abstract

Ram semen cryotolerance problem continues despite intensive research. This study aimed to evaluate the effect of replacing total seminal plasma (SP) with that of high‐cryotolerant rams or extender on semen quality parameters in low‐cryotolerant rams. Rams previously determined as low (*n* = 6) and high‐cryotolerant (*n* = 5) were used in the breeding season. High‐cryotolerant rams were only included with their SP in the SP+ group. Each normospermic fresh ejaculate from low‐cryotolerant rams was split into three aliquots to form the groups. The control aliquot was diluted using a two‐step process. Centrifugation removed SP from the second aliquot (SP− group), and the sperm pellet was rediluted with a Tris‐based extender using the same amount of SP removed. The third aliquot's SP was removed as in the SP− group, but the remaining sperm pellet was rediluted with fresh SP harvested from high‐cryotolerant rams using the same amounts of removed SP in the SP+ group. Semen was rediluted with a Tris‐based extender in the SP− and SP+ groups using a two‐step process as in the control group. Sperm quality was similar between the groups after dilution and equilibration. However, the post‐thaw semen quality was higher in the SP− (one parameter) and SP+ (four parameters) groups than in the control group (*p* < 0.05). Additionally, these parameters were similar in the SP− and SP+ groups. This study showed that, although an adequate extender had positive effects, replacing the whole SP with that of high‐cryotolerant rams may better solve the low cryotolerance problem in ram semen.

## Graphical abstract

Altering the seminal plasma ingredients by replacement, especially with seminal plasma from that of high‐cryotolerant rams, can be recommended as an alternative method to increase cryotolerance in rams with low cryotolerance.

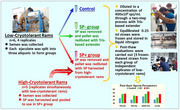

## 1 Introduction

Artificial insemination (AI) is a crucial assisted reproductive technology in livestock genetic improvement and reproductive management (Alvares et al. 2015). It enables the most effective use of genetically superior males (Faigl et al. 2012). It is also a good way of preventing venereal diseases in livestock (Gibbons et al. 2019). Besides this, preserving the semen by freezing is the most important requirement to benefit from these advantages. However, frozen‐thawed semen usage in sheep is still limited due to poor fertility results (Alvarez et al. 2019). The freeze‐thawing process changes biological and functional events, alters sperm acrosome, mitochondria, and plasma membrane functional integrity, and decreases fertilizing ability (Sharafi et al. 2022). Many factors cause a decrease in the fertility of frozen‐thawed ram semen, and intensive studies are still being carried out to overcome this trouble (Vozaf et al. 2022).

As a complex fluid, seminal plasma (SP) originates from the male reproductive canal. It may have both inhibition and stimulation effects on sperm function owing to the multifunctional actions of its ingredients, such as minerals, antioxidants, salts, sugars, and a huge number of various proteins (Agarwal et al. 2014; Akalın et al. 2015; Carvajal‐Serna et al. 2018; Wu et al. 2020; Hitit et al. 2021; Chen et al. 2024). All of these organic and inorganic constituents have known and/or unknown functions affecting the quality of fresh and cryopreserved semen (Ben moula and El Amiri 2022; Dayanıklı et al. 2022). Critical positive effects of these ingredients on sperm function regulation occur via stabilization and increase of the sperm membrane resistance during storage and cold shock stress (Colás et al. 2009) and via reduction of freeze‐thawing harmful effects (Bernardini et al. 2011). However, the presence, absence, or differences in SP enzyme and protein content may modify these beneficial effects (Agarwal et al. 2014). Hitit et al. (2021) determined differences in the abundance of diverse proteins in the high and low fertile Merino rams’ semen. Specifically, some proteins (Glutathione s‐transferase mu 5 (GSTM5), heat shock protein 90 (HSP90), 26S proteasome complex (PSMD13), the TCP‐1 complex (CCT), sorbitol dehydrogenase (SORD), angiogenin‐2‐like protein (ANG), zinc‐2‐alpha glycoprotein (AZGP1), cystatin (CST3), cathepsin B (CTSB), RSVP14, RSVP20, RSVP22, and AZGP1) (Fernández‐Juan et al. 2006; Ledesma et al. 2014; Rickard et al. 2015; Rickard et al. 2016; Ryu et al. 2019) affect semen cryotolerance via acting as sperm motility activators and intracellular modulators (Qu et al. 2007) and/or regulators of the cyclic AMP (cAMP)/cAMP‐dependant protein kinase A (PKA) signalling pathway (Brokaw 1987). There are also only few genomic and proteomic studies that reveal some single‐nucleotide polymorphisms (SNP) (Bülbül et al. 2025) or proteins (voltage‐dependent anion‐selective channel protein 2 [VDAC2], Voltage‐gated cation channel activity, high voltage–gated calcium channel activity, voltage–gated calcium channel activity, cAMP binding, intracellular cyclic nucleotide–activated cation channel activity, intracellular cAMP–activated cation channel activity, and cyclic nucleotide–gated ion channel activity) effect on semen cryotolerance (Ryu et al. 2019; Bülbül et al. 2025) via influencing ion transport (Hinsch et al. 2004). These proteins regulate the plasma membrane potential. They protect spermatozoa by stabilizing the plasma membrane and providing intracellular and intramembrane ionic concentration maintenance. The ionic content reduces cold and osmotic shock damage, minimizing cryoinjury to the plasma membrane and the DNA structure (Rizkallah et al. 2022; Saha et al. 2022).

In normal mating, the sperm cell is exposed to SP for a relatively short time. However, it is subjected to SP for longer in cryopreservation procedures, which may alter the SP effect on spermatozoa (Burroughs et al. 2013). In fact, the SP effect on cryopreserved semen fertility is controversial (Ben moula and El Amiri 2022). Reports are indicating the addition of SP decreased (de Graaf et al. 2007), improved (Bernardini et al. 2011), or did not affect (Ledesma et al. 2015) semen quality both before and after semen freeze‐thawing. In addition, insemination using frozen‐thawed ram semen resulted in conflicting pregnancy results in which SP increased (de Graaf et al. 2007), did not affect (O'Meara et al. 2007), or affected in an inconsistent manner (Leahy et al. 2010). Even studies report that SP may be toxic (via its zinc and arsenic content) (de Graaf et al. 2007; Wu et al. 2020; Chen et al. 2024) or non‐toxic (Leahy et al. 2009) to spermatozoa. Besides all these, recent studies have been carried out to overcome possible adverse effects of SP. In their studies, researchers proved that supplementing the fresh ram ejaculates with 5% (Green et al. 2020) or 12% (Rickard et al. 2016) SP from superior or inferior ejaculates can increase or decrease cryopreserved sperm motility, respectively. It has also been reported that rediluting SP‐removed bull ejaculate with a Tris‐based extender (TRIS buffer + 6% glycerol + 20% egg yolk) resulted in higher post‐thaw semen quality (Steinhauser et al. 2016). Egg yolk and trehalose have cryoprotective effects on semen (Pelufo et al. 2015; Rajabi‐Toustani et al. 2021). Phospholipids in egg yolk replace lost or damaged sperm membrane phospholipids (Graham and Foote 1987) and form a protective film on the surface of the sperm (Quinn et al. 1980) in the freeze‐thawing process. Trehalose shows its effect by forming hydrogen bridges with the plasma membrane phospholipids. It prevents intracellular ice crystal formation by replacing water molecules (Bakás and Disalvo 1991). Steinhauser et al. (2016) obtained better post‐thaw semen quality by removing the SP from bull ejaculate by centrifugation and rediluting the remaining pellet with a Tris‐based extender.

In a previous study conducted by our team 2 weeks prior to this experiment, the putative genetic functional cryotolerance of the rams was determined to be low (Hasak) and high (Hasmer) (Bülbül et al. 2025). Although some researchers have replaced SP in different animal species or different proportions in rams, to the best of our knowledge, this is the first report evaluating the effect of replacing total SP with that of high‐cryotolerant rams or extender on semen quality parameters in low‐cryotolerant rams. Therefore, the aim of this study was to evaluate the effect of the replacement of total SP with that of high‐cryotolerant rams or extender on semen quality parameters in low‐cryotolerant rams.

## 2 Materials and Methods

All procedures in this study were compatible with the Animal Experiments Local Ethics Committee guidelines of Bahri Dağdaş International Agricultural Research Institute, Turkey (30.11.2020/114). ARRIVE guidelines were also adhered to by the authors.

### 2.1 Materials

The study was carried out in the breeding season. Fertility‐proven 11 rams (2.5–4.5 years old, 6 Hasak and 5 Hasmer, crosses of German Black Head, Hampshire Down, Akkaraman, and Anatolian Merino breeds) were kept indoors at the Sheep Breeding Research Institute, Balıkesir, Turkey (128 m above sea level, 40°19′43″ latitude and 27°54′27″ longitude). The ambient temperature and average relative humidity were 19°C–32°C and 65%, respectively. Animals were kept under standardized lighting and housing conditions and fed according to the guidelines of the National Research Council (2007). Additional supplements to satisfy the rams’ nutritional requirements and water were supplied ad libitum.

### 2.2 Methods

The putative genetic functional cryotolerance of the rams was determined as low (Hasak) and high (Hasmer) in our previous study conducted 2 weeks prior to this experiment during the breeding season (Bülbül et al. 2025). Roughly, although the quality parameters were similar in Hasak and Hasmer rams for both fresh semen (total and progressive motility [PM], *p *> 0.6) and after equilibration (total and PM, live spermatozoa, and abnormal morphology, *p* > 0.1), the post‐thaw semen traits, including total motility (*p* = 0.002), PM (*p* = 0.001), live spermatozoa (*p* = 0.002), live spermatozoa with intact acrosome (*p* = 0.002), dead spermatozoa (*p* = 0.002), and dead spermatozoa with damaged acrosome (*p* = 0.016), were superior in rams with high cryotolerance compared to those with low cryotolerance (Bülbül et al. 2025).

#### 2.2.1 Semen Collection and Evaluation

In this experiment, although the experimental groups (Control, SP− and SP+) were created with Hasak rams (*n* = 6), Hasmer rams (*n* = 5) were only included with their SP in the relevant group (SP+). An artificial vagina was used to collect semen following standard procedures (Paulenz et al. 2002), with four replicates in total every other day. After measuring the volume using a 0.1‐mL graduated conical tube, the ejaculates were immediately transferred to a water bath (30°C). First, the mass activity and motility of fresh semen were examined via a phase‐contrast microscope (Olympus Corporation, CX31, Japan) at 40× magnification. A sperm density meter was used to determine sperm concentration (IMV Technologies, Ovine AccucellR, France). Normospermic ejaculates (volume > 0.9 mL, concentration > 1800 × 10^6^/mL, mass movement > 4 (1–5), and total motility ≥ 70%) were used to form the groups (Galarza et al. 2020).

#### 2.2.2 Semen Processing and Forming the Groups

**FIGURE 1:**
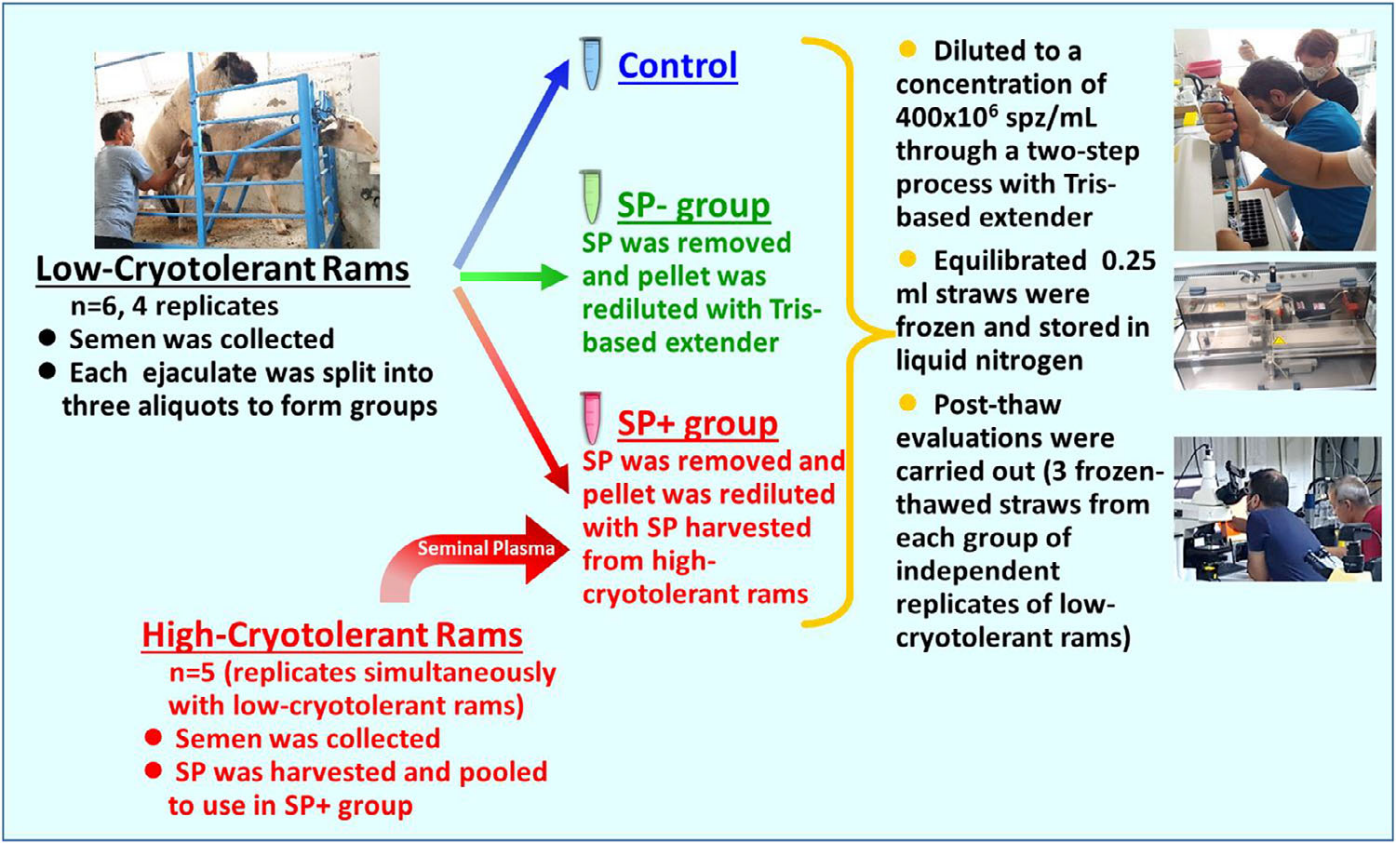
The study design. SP, seminal plasma.

The study design is illustrated in Figure 1. After collection and initial evaluation, each suitable fresh ejaculate was split into three aliquots to form the study groups. The first aliquot was accepted as the control and diluted to a concentration of 400 × 10^6^ spz/mL through a two‐step dilution process (Nur et al. 2010) with a Tris‐based extender (Tris 2.71 g Sigma T6791, Citric acid 1.0 g Sigma C2404, d‐Fructose 1.4 g Sigma F3510, %20 egg yolk + Trehalose 50 mM Sigma T0167) (Aisen et al. 2000).

The second aliquot was processed to form the SP− group. First, SP was removed by splitting into Eppendorf tubes to contain approximately 600 × 10^6^ spermatozoa, adding 1 mL Tris‐based extender to each Eppendorf, and centrifuging at 700 × *g* for 10 min at room temperature (Ustuner et al. 2018; Rajabi‐Toustani et al. 2021). Following the centrifugation, the supernatant was immediately taken away, and the remaining sperm pellet was rediluted using a Tris‐based extender with the same volume of previously removed SP. The semen rediluted with a Tris‐based extender was then diluted to a final concentration of 400 × 10^6^ spz/mL using a two‐step dilution process as in the control group.

The third aliquot was used to form the SP+ group, where the SP was removed similarly to the SP− group. The remaining sperm pellet was then re‐suspended with fresh SP (SP from normospermic ejaculates) harvested from high‐cryotolerant rams using the same volume of SP previously removed. This semen with the replaced SP was subsequently diluted to a final concentration of 400 × 10^6^ spz/mL using a two‐step dilution process as in the control group.

#### 2.2.3 SP Collection

SP was harvested from high‐cryotolerant Hasmer rams at the same time as low‐cryotolerant Hasak rams, which formed the groups. After collection of the ejaculates from Hasmer rams, the initial evaluation was completed immediately, and normospermic ejaculates were centrifuged at 6500 × *g* for 15 min at 4°C. Following the centrifugation, the SP was harvested, filtered (cat# 094.08.006, ISOLAB GmbH syringe filter with 0.22 µm pore size, Germany) and pooled for redilution of the semen in group SP+ (Rajabi‐Toustani et al. 2021).

#### 2.2.4 Cryopreservation and Thawing

After the dilution process, all semen from all groups was equilibrated at 5°C for 2 h and loaded into straws (0.25 mL, Minitüb GmBH, Tiefenbach, Germany). Straws were frozen in liquid nitrogen vapour using a programmable freezer (Mini‐Digitcool IMV, France) according to the protocol recommended by IMV (5°C/min between 5°C and −10°C, 80°C/min between −10°C and −55°C, 40°C/min between −55°C and −100°C, 20°C/min between −100°C and −140°C, totally 6.5 min). After freezing, the straws were stored in liquid nitrogen (−196°C) for at least 1 week until thawed. The post‐thaw evaluations were carried out using at least three thawed straws (in a water bath, 30 s at 37°C) from each group of independent replicates (Şengül et al. 2024).

#### 2.2.5 Semen Traits’ Evaluation

All semen samples (fresh, equilibrated, or frozen‐thawed) were diluted using a Tris‐based egg yolk‐free extender at a ratio of 16 × 10^6^ spz/mL to prevent failure caused by over‐concentration and to ensure uniformity of the method for motility, morphology, and flow cytometry analyses.

##### 2.2.5.1 Sperm motility

Total (TM) and PM, rapid, medium, and slow sperms (%), head area (µm^2^), and the kinematic parameters (curvilinear velocity (VCL) (µm/s), rectilinear velocity (VSL) (µm/s), linearity (LIN) (%), average path velocity (VAP) (µm/s), wobble (WOB) (%), straightness (STR) (%), amplitude of lateral head displacement (ALH) (µm) and beat‐cross frequency (BCF) (Hz)) were identified via a computer‐assisted sperm analyser (CASA) (SCA, Version 6.5.090, Microoptics, Spain) (Galarza et al. 2020). Shortly, a 3 µL semen sample was placed on a slide (Leja, Ref. 025107, IMV Technologies, France) specific for the CASA system. Sperm movement characteristics were evaluated at field settings: min–max 15–70 µm^2^, speed settings: set to static (<10 µm/s), slow to medium (>45 µm/s), fast (>75 µm/s) and progressive (STR > 80). The analysis was fulfilled when at least 500 spermatozoa or at least 7 areas were analysed.

##### 2.2.5.2 Sperm morphology

Morphological examinations were carried out using the Giemsa staining protocol (Hafez 2000). For this aim, a saline solution drop and a diluted semen drop were placed on a heated slide and then smeared and dried in air. The smears were fixed for 10 min in methyl alcohol and stained in Giemsa dye cuvettes for 50 min. The light microscope's immersion objective (×100) (Eclipse E200, Nikon, Japan) was used to count 200 spermatozoa from each sample. The number of spermatozoa having morphological defects (acrosome, head, middle, and tail) was determined.

##### 2.2.5.3 Flow cytometry analyses

Functional and structural integrity analyses of sperm were performed using the Guava easyCyte device (containing a single blue laser [488 nm], two photodiodes [forward scatter and side scatter], three photo multiplicators [green: 525/30 nm, red: 655/50 nm and yellow: 583/26 nm], and suitable optical filters and brackets) and the CytoSoft programme (Guava Technologies Inc., Hayward, CA, USA). Instrument accuracy was checked daily via Guava Check kit (Guava Technologies, Inc., Millipore, Billerica, MA, USA). Every analysis was finished by counting 5000 spermatozoa having scatter and fluorescent properties.

###### 2.2.5.3.1 Viability and acrosome integrity

The acrosome integrity and viability of spermatozoa were analysed using an Easykit 5 kit (IMV Technologies, cat# 025293) in accordance with the IMV recommendations. Shortly, 200 µL Easy Buffer B (IMV Technologies, cat# 019449) and 5 µL of TRIS diluted semen (approximately 40,000 spermatozoa) were added to a ready‐to‐use 96 well plate and were incubated for 45 min at 37°C in the dark. The ratio of spermatozoa that have intact/damaged plasma membrane and acrosome was calculated via the software programme (IMV Technologies, EasySoft, Ref. 024842) (Battut et al. 2016).

###### 2.2.5.3.2 Mitochondrial Membrane Potential (MMP)

Sperm MMP (%) was analysed using EasyKit 2 (IMV Technologies, Ref. 024864) following the manufacturer's protocol. Briefly, the fluorochromes were dissolved by adding 10 µL of ethanol to the 96 ready‐to‐use wells. Then, 190 µL of Easybuffer B (IMV Technologies, Ref. 023862) and 5 µL of tris diluted semen (50,000 spermatozoa approximately) were added and incubated in the dark at 37°C for 30 min. The spermatozoa with high fluorochrome concentration were matched with polarized mitochondria (high Δψm), while mitochondria accumulated lower fluorochrome concentration (low Δψm) were calculated as depolarized. The software programme (IMV Technologies, EasySoft, Ref. 024842) calculated the polarized/ depolarized semen ratio (Battut et al. 2016).

#### 2.2.6 Statistical Analyses

All sperm and kinematic parameters were presented as *X* ± standard error mean. The obtained data were analysed using one‐way analysis of variance (ANOVA) after homogeneity and normality checks were done via Levene's test and Kolmogorov‐Smirnov test, respectively. The Tukey comparison method was used to compare the group differences. The significance level was considered as *p* ≤ 0.05. All statistical analyses were completed using a computer programme (IBM SPSS Statistics for Windows, Version 23). The sample size (6 rams × 4 replicates × 3 straws = 72 samples) was higher than the minimum of 66 samples calculated using the programme GPower 3.1 (http://www.gpower.hhu.de/). The mean of the three groups was measured using one‐way ANOVA test with 95% power, 50% effect size, and 0.05 Type 1 error (Faul et al. 2007).

## 3 Results

**FIGURE 2:**
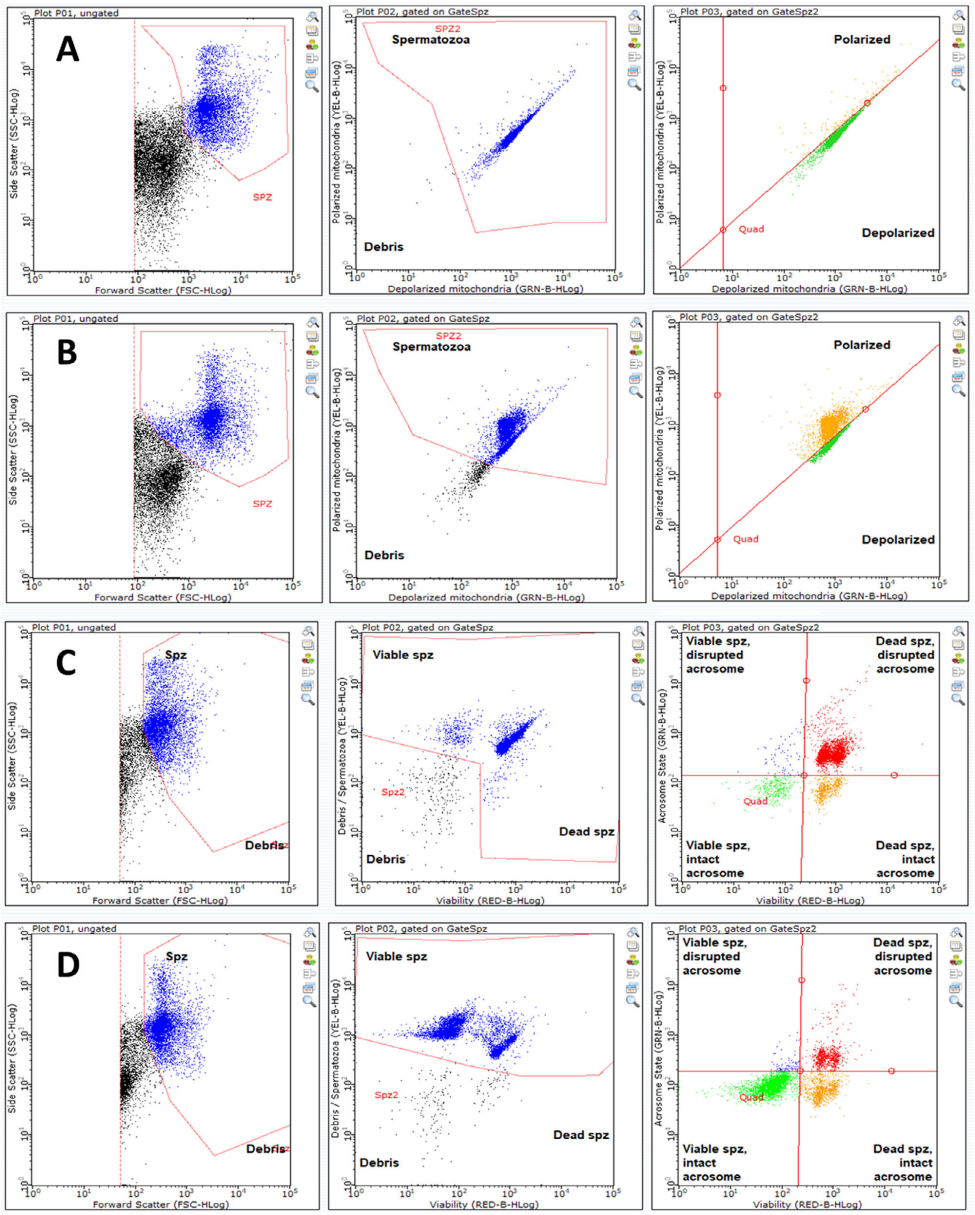
Examples of flow cytometer analyses results. (A) An example of the dotplots from flow cytometry analysis in the control group. Flow cytometry analysis of an experiment showing cells sorted dotplots using Easy kit‐2 (mitochondrial activity). This kit contains JC‐1 dye, a substance recognised for its ability to distinguish between polarised and depolarised mitochondria by exhibiting a visible orange/red or green colour, respectively. The spermatozoa with high fluorochrome concentration were matched with polarized mitochondria (high Δψm). At the right dotplot; orange population; polarized mitochondria/high mitochondrial potential, green population; depolarized mitochondria/low mitochondrial potential. (B) An example of the dotplots from flow cytometry analysis in the SP+ group. Flow cytometry analysis of an experiment showing cells sorted dotplots using Easy kit‐2 (mitochondrial activity). The spermatozoa with high fluorochrome concentration were matched with polarized mitochondria (high Δψm). At the right dotplot; orange population; polarized mitochondria/high mitochondrial potential, green population; depolarized mitochondria/low mitochondrial potential. (C) An example of dotplots from flow cytometry analysis in the control group. Flow cytometry analysis of an experiment showing cells sorted dotplots using Easy kit‐5 (viability & acrosome integrity). This kit allows the simultaneous assessment of damaged acrosomes in both live and dead sperm cells by incorporating peanut agglutinin (PNA), a cell‐permeable orange fluorescent nucleic acid stain known as Syto83 and propidium iodide (PI) fluorescent stains. At the right dotplot; green population: Viable and intact acrosome, orange population: dead and intact acrosome, red population: dead and damaged acrosome, blue dots: viable but damaged acrosome. (D) An example of the dotplots from flow cytometry analysis in the SP+ group. Flow cytometry analysis of an experiment showing cells sorted dotplots using Easy kit‐5 (viability & acrosome integrity). At the right dotplot; green population: viable and intact acrosome, orange population: dead and intact acrosome, red population: dead and damaged acrosome, blue dots: viable but damaged acrosome.

The difference in spermatological parameters between the study groups after dilution (TM and PM) and equilibration (TM, PM, MMP, and live and dead spermatozoa) was insignificant (*p* > 0.05) (Table 1). Post‐thaw TM, PM, MMP, and live spermatozoa rates (%) were higher, whereas dead spermatozoa rates (%) were lower in SP− and SP+ groups than in the control group (*p* < 0.05). However, these parameters were similar in the SP− and SP+ groups. Examples of flow cytometric analyses were given in Figure 2.

**TABLE 1:** Spermatological parameters after dilution and equilibration and post‐thaw in groups (Mean ± SEM).

Parameters	Control	SP−	SP+	*p* value
**After dilution**				
TM (%)	85.08 ± 2.28	87.01 ± 1.47	87.86 ± 1.33	0.517
PM (%)	69.93 ± 2.92	67.62 ± 3.01	67.24 ± 3.45	0.807
**After equilibration**				
TM (%)	91.44 ± 1.13	91.84 ± 0.61	89.75 ± 1.40	0.368
PM (%)	81.32 ± 1.56	77.99 ± 1.65	80.64 ± 1.94	0.359
MMP (%)	70.37 ± 2.95	67.99 ± 3.14	64.26 ± 2.83	0.349
Live spermatozoa (%)	71.95 ± 1.52	70.27 ± 1.60	69.34 ± 1.97	0.550
Intact acrosome (%)	70.54 ± 1.54	69.08 ± 1.58	68.03 ± 1.93	0.576
Damaged acrosome (%)	1.41 ± 0.13	1.19 ± 0.10	1.31 ± 0.12	0.392
Dead spermatozoa (%)	28.05 ± 1.53	29.73 ± 1.60	30.66 ± 1.97	0.551
Intact acrosome (%)	18.87 ± 0.95	17.87 ± 0.93	18.80 ± 1.48	0.790
Damaged acrosome (%)	9.18 ± 0.86	11.86 ± 0.96	11.86 ± 0.83	0.055
Abnormal morphology (%)	5.99 ± 1.34	3.83 ± 0.41	3.78 ± 0.51	0.124
**Post‐thaw**				
TM (%)	24.11 ± 3.55^b^	36.56 ± 3.86^ab^	37.53 ± 4.04^a^	**0.026**
PM (%)	20.09 ± 3.15^b^	31.98 ± 3.49^a^	30.73 ± 3.64^ab^	**0.032**
MMP (%)	29.68 ± 2.85^b^	36.83 ± 3.08^ab^	43.56 ± 4.06^a^	**0.018**
Live spermatozoa (%)	20.53 ± 2.82^b^	26.31 ± 2.51^ab^	36.22 ± 3.57^a^	**0.002**
Intact acrosome (%)	19.49 ± 2.75^b^	25.13 ± 2.47^ab^	33.93 ± 3.47^a^	**0.003**
Damaged acrosome (%)	1.04 ± 0.17^b^	1.18 ± 0.18^b^	2.28 ± 0.30^a^	**0.000**
Dead spermatozoa (%)	79.47 ± 2.82^a^	73.69 ± 2.51^ab^	63.78 ± 3.57^b^	**0.002**
Intact acrosome (%)	34.56 ± 2.66	36.25 ± 2.95	31.09 ± 2.95	0.433
Damaged acrosome (%)	44.91 ± 4.65	37.43 ± 4.14	32.69 ± 4.01	0.133
Abnormal morphology (%)	8.59 ± 0.75	8.07 ± 0.72	8.00 ± 0.86	0.843

*Note*: Differences between means indicated by different letters (a, b) in the same row are significant (*p* < 0.05) (indicated in bold).

Abbreviations: MMP, mitochondrial membrane potential; PM, progressive motility; TM, total motility.

**FIGURE 3:**
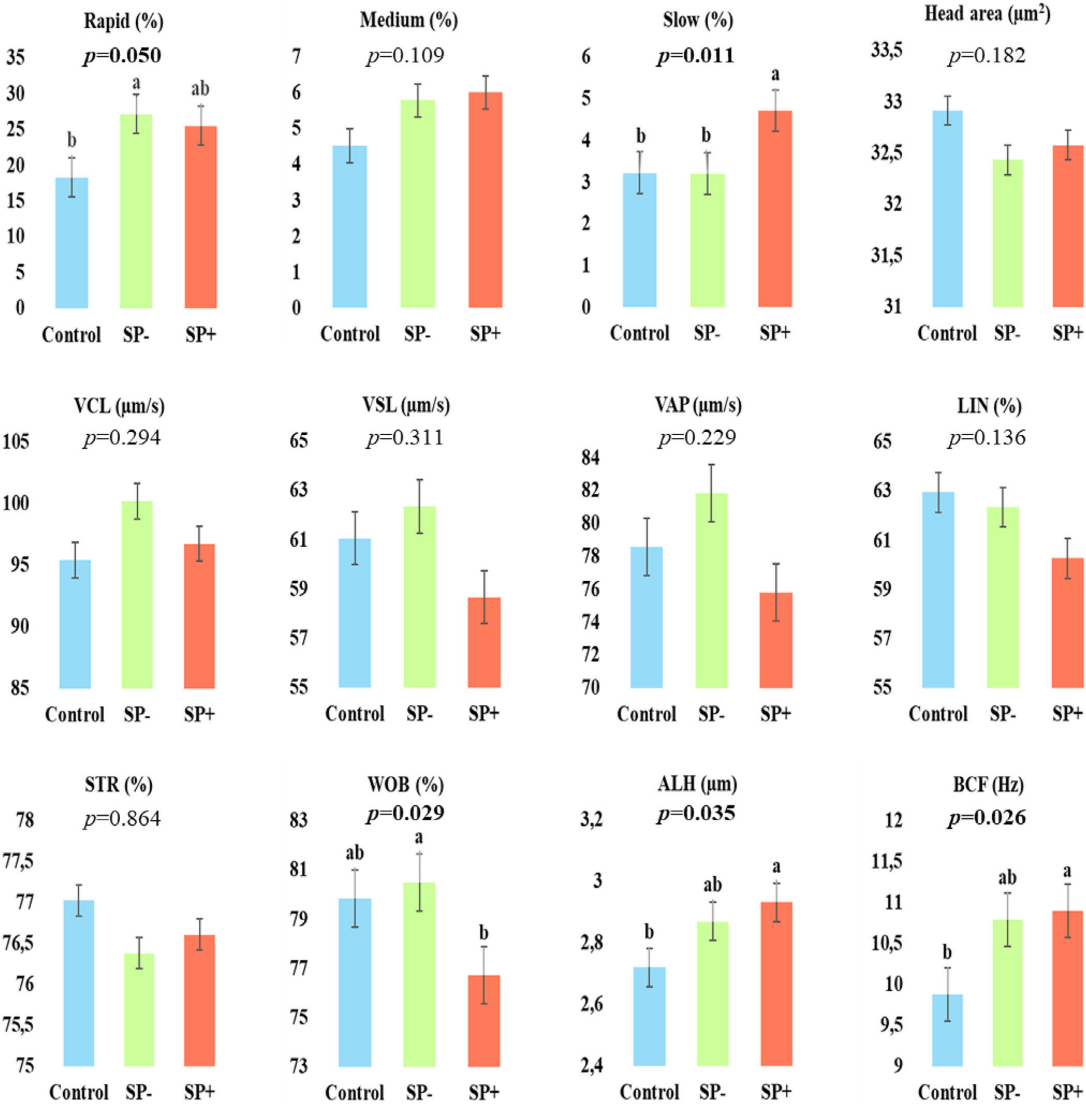
Post‐thaw kinematic parameters of spermatozoa in groups (±SEM). ^a,b^: The differences between means with different superscripts are significant (*p* ≤ 0.05). ALH, amplitude of lateral head displacement (µm); BCF, beat‐cross frequency (Hz); LIN, linearity (%); SP, seminal plasma; STR, straightness (%); VAP, average path velocity (µm/s); VCL, curvilinear velocity (µm/s); VSL, rectilinear velocity (µm/s); WOB: wobble (%).

The post‐thaw spermatological kinematic parameters in the groups were presented in Figure 3. The differences between groups were significant for rapid, slow, WOB, ALH, and BCF parameters (*p* ≤ 0.05). Although the difference in only one parameter was significant between control and SP− groups (rapid), the difference in three parameters was significant between control and SP+ groups (slow, ALH, and BCF).

## 4 Discussion

This study showed that an adequate extender had positive effects, but replacing the whole SP with that of high‐cryotolerant rams may better solve the problem of low cryotolerance in ram semen. Spermatological parameters were similar in all groups after dilution and equilibration. There was a post‐thaw improvement in all parameters in the SP− group compared to the control group, but the differences were insignificant except for PM. However, semen quality in the SP+ group was superior to that of the control group for all parameters except for PM. In addition, the post‐thaw semen quality was similar in the SP− and SP+ groups.

There are many studies demonstrating cryotolerance differences between individual animals in bulls (Jobim et al. 2004), stallions (Loomis et al. 2008), and rams (Rickard et al. 2016). Similarly, we got the same result in our previous study (Bülbül et al. 2025). Although the quality was similar for both fresh semen (total and PM, *p* > 0.6) and after equilibration (total and PM, live spermatozoa, and abnormal morphology, *p* > 0.1), the post‐thaw semen traits, including total motility (29.7 vs. 45.1%, *p* = 0.002), PM (23.2 vs. 35.8%, *p* = 0.001), live spermatozoa (26.5 vs. 40.7%, *p* = 0.002), live spermatozoa with intact acrosome (25.6 vs. 39.7%, *p* = 0.002), dead spermatozoa (73.5 vs. 59.3%, *p* = 0.002) and dead spermatozoa with damaged acrosome (41.3 vs. 28.4%, *p* = 0.016), were superior in rams with high cryotolerance than those with low cryotolerance (Bülbül et al. 2025). In previous studies, even when the same method and diluents were used in semen freezing, breed (Tekin et al. 2006; Vozaf et al. 2022) and individual (Vozaf et al. 2022) differences in the negative effects of ram semen cryopreservation were also observed. The causes of individual variations are described as nutrition, season, age, breed and some other unknown factors that alter sperm membranes or SP composition (Green et al. 2020; Ben moula and El Amiri 2022; Sharafi et al. 2022; Vozaf et al. 2022). Whatever the reason, SP composition variation is one of the most important responsible ones for the cryotolerance variation between rams (Rickard et al. 2016). Hence, although there are studies documenting the positive effect of SP during the freezing process in ram (Leahy et al. 2010), bull (Almadaly et al. 2015) and boar (Vadnais and Althouse 2011), detrimental effects of SP after freezing‐thawing also have been reported in ovine (de Graaf et al. 2007) and porcine (Fernández‐Gago et al. 2010) sperm. SP, as a complex medium, contains a huge variety of organic and inorganic contents. This wide variety of ingredients, such as minerals, antioxidants, salts, sugars, and a great number of various proteins, may have cons and/or pros on semen quality and cryotolerance (Rickard et al. 2016; Carvajal‐Serna et al. 2018; Almadaly et al. 2021). However, as the most significant component by weight, proteins may be more effective than others on sperm function (Leahy et al. 2012). Differences were also detected in the abundance of various proteins in the semen of relatively more and less fertile Merino rams (Hitit et al. 2021). Moreover, Rickard et al. (2016) determined significant proteomic differences concerning the abundance of specific SP proteins between SP collected from rams with low or high cryotolerance. A freezability biomarker GSTM5 was at different levels in bull sperm with high and low freezing tolerance (Ryu et al. 2019). Another study by Rickard et al. (2015) demonstrated that some proteins (HSP90, PSMD13, CCT, and SORD) were correlated with high cryotolerance, whereas others (ANG, AZGP1, CST3 and CTSB) were correlated with low cryotolerance. SP's beneficial effect mainly originates from proteins with low molecular weight (≤30 kDa) and is reported to be concentration‐dependent. Some SP proteins, such as RSVP14 (Fernández‐Juan et al. 2006; Ledesma et al. 2014), RSVP20 (Fernández‐Juan et al. 2006) and RSVP22 (Ledesma et al. 2014) with low molecular weight, were stated to reduce cryodamage on sperm. A prostate‐secreted protein AZGP1 was previously shown to be a sperm motility activator and intracellular modulator (Qu et al. 2007) functioning by upregulating the cAMP/cAMP‐dependant PKA signalling pathway (Brokaw 1987). That is, the alteration of SP protein content may change ionic balance, which may have positive or negative effects on sperm function (Sharafi et al. 2022). Rickard et al. (2016) proved that supplementing the fresh ram ejaculates with %12 SP from high‐ or poor‐quality ejaculates can increase or decrease cryopreserved sperm motility, respectively. Green et al. (2020) also demonstrated that motility is determined by SP composition changes and got the same conclusion by supplementing the fresh ram ejaculates with %5 SP.

Few genomic and proteomic studies unveiling some SNPs (Bülbül et al. 2025) or proteins (Ryu et al. 2019; Bülbül et al. 2025) affecting semen cryotolerance have been conducted. In addition to the direct effects of protein content on semen quality as reviewed above, some proteins such as VDAC2 (Ryu et al. 2019), a mitochondrial protein, have a direct effect on cryopreservation success via its role in ion transport (Hinsch et al. 2004). Bülbül et al. (2025) also revealed the relationship between some ion channels (voltage‐gated cation channel activity, high voltage–gated calcium channel activity, voltage‐gated calcium channel activity, cAMP binding, intracellular cyclic nucleotide–activated cation channel activity, intracellular cAMP‐activated cation channel activity, and cyclic nucleotide–gated ion channel activity) and cryotolerance. Many ions such as calcium (Ca^2+^), potassium (K^+^), bicarbonate (HCO_3_^−^), chloride (Cl^−^), sodium (Na^+^) and hydrogen (H^+^) have crucial impacts on semen fertility via their roles in regulating plasma membrane potential. They are also important in sperm adaptation to the surrounding medium. These ions perform their functions through specialized ion channels located on the cell membrane, which allow them to pass from the outside to the inside of the sperm cell (Puga Molina et al. 2018; Yeste et al. 2020; Noto et al. 2021; Rodríguez‐Páez et al. 2021). The effects of SP and the extender ionic ingredients on semen cryopreservation are well known. They protect spermatozoa by stabilizing the plasma membrane and providing intracellular and intramembrane ionic concentration maintenance. Moreover, thanks to their ionic structure, they reduce cold and osmotic shock damages and minimize cryoinjuries on the plasma membrane and DNA structure (Rizkallah et al. 2022; Saha et al. 2022). However, the ionic balance of the SP and media is crucial. In this context, Sharafi et al. (2022) reviewed some of the possible damaging effects of ionic components during freeze‐thawing and the likely reducing effects of cryoprotectants (such as trehalose, as in this study) against these harmful effects. However, genomic, proteomic or metabolomic studies are still needed in this field.

Ram fertility is crucial in sheep farming as a single ram can produce hundreds of lambs a year. Semen quality directly affects the number of insemination doses derived from each ejaculate (Dayanıklı et al. 2022). However, poor fertility results still limit frozen‐thawed semen usage in sheep (Alvarez et al. 2019). Solving the low cryotolerance problem may allow wider use of AI, leading to more effective and feasible use of genetically superior males (Faigl et al. 2012). The importance of the extender for semen cryopreservation is well known. Egg yolk, especially, as a non‐penetrating cryoprotectant, protects sperm successfully against the negative effects of freeze‐thawing (Bathgate et al. 2006; Bispo et al. 2011; García et al. 2017; Rajabi‐Toustani et al. 2021). It was determined that sperm washing by centrifugation positively affected frozen‐thawed ram semen quality (García et al. 2017). There are also semen cryopreservation studies that showing the protective effect of extenders containing egg yolk after removing the SP against the cold shock in boar (Bathgate et al. 2006) or buck (Bispo et al. 2011). Egg yolk phospholipids in LDL also protect spermatozoa by replacing lost or damaged sperm membrane phospholipids during the freeze‐thawing (Graham and Foote 1987), creating a protective film on the surface of the sperm (Quinn et al. 1980), and competing with harmful SP cationic peptides (<5 kDa) for sperm membrane binding (Vishwanath et al. 1992). García et al. (2017) suggested a negative interaction between SP and egg yolk. In another study, Marco‐Jimenez et al. (2004) determined that the egg yolk content of the extender changed the sperm VCL, VSL, and LIN. The researchers suggested that the gelatinous consistency of the extender, which may be responsible for the difference, is changed by its ingredients and the egg yolk content (Marco‐Jimenez et al. 2004). Extenders, including trehalose, have also been used as cryoprotectants in ram with successful results (Bucak et al. 2007; Pelufo et al. 2015). The cryoprotective effect of trehalose originates from its hydrogen bridges forming with the plasma membrane phospholipids. Thus, it replaces water molecules and prevents intracellular ice crystal formation (Bakás and Disalvo 1991). Bakás and Disalvo (1991) also reported that the cryoprotective effect of trehalose is inhibited by Ca^2+^ because of the competitive effect between each other. Steinhauser et al. (2016) removed SP from bull ejaculate by centrifugation with a Tris‐based extender and rediluted the remaining pellet using 0% or 10% SP (the rest with TRIS buffer + 6% glycerol + 20% egg yolk). They determined that semen samples containing 0% SP had better post‐thaw quality (live‐oriented cells, dead sperm, and motility). They also suggested that removing the acrosomal enzymes released from dead spermatozoa may be one of the reasons for the higher semen quality (Steinhauser et al. 2016).

There is also a wide variation in SP content between animal species (Sharafi et al. 2022). Regarding this variety, there are recent studies that used SP from different species in the freezing extender to minimize the cryodamage effect on spermatozoa. Improved post‐thaw quality was obtained after removing buck semen SP and then rediluting it with 5% bovine SP‐supplemented egg yolk (7.5% egg yolk)–Tris extender (Dhara et al. 2023). Alcay et al. (2020) demonstrated the positive effects of rainbow trout SP‐supplemented lecithin‐based extenders on goat spermatozoa. Better post‐thaw quality was also reported using egg yolk extender supplemented with 10% and 1% rainbow trout SP in ram semen (Ustuner et al. 2016).

Although there was a post‐thaw improvement in all parameters in the SP− group compared to the control group, the differences were insignificant except for PM in our study. However, the semen quality in the SP+ group was superior to that of the control group in all parameters except for PM. In addition, the post‐thaw semen quality was similar in the SP− and SP+ groups. Our study did not determine the SP protein and ion contents of high and low‐cryotolerant semen. It could be suggested that some of these proteins and/or ions may be responsible for the semen quality results in our study. Replacing SP with that from high‐cryotolerant rams may have compensated for possible adverse effects caused by proteins, ions, and/or some unknown factors. Another possible reason for the improved semen quality by replacing the SP with an extender may be the positive effects of egg yolk and cryoprotectants in the replaced extender. That is, more studies are needed to specify the molecular mechanisms in SP composition that cause this difference in our hypothesis‐generating study. In addition, our positive findings obtained by replacing the low‐cryotolerant ram SP with that of a different breed may be considered a limitation of this study due to the probable breed‐related differences in SP as discussed above, and further research is recommended.

## 5 Conclusions

Although the mechanisms have not yet been fully understood, altering the SP ingredients by replacement, especially with SP from that of high‐cryotolerant rams, can be recommended as an alternative method to increase cryotolerance in rams with low cryotolerance. These findings may contribute to solving the problem of cryopreservation and improving reproduction in animal species. Further in vivo studies are also needed in this field.

## Author Contributions

Cemal Dayanıklı: writing – review and editing, writing – original draft, conceptualization, methodology, investigation, data curation. **Bülent Bülbül**: writing – review and editing, writing – original draft, conceptualization, methodology, investigation, data curation, visualization. **Şükrü Doğan**: writing – review and editing, conceptualization, formal analysis, investigation, project administration. **Ebru Şengül**: writing – review and editing, investigation, data curation. Mesut Kırbaş: writing – review and editing, investigation, data curation. **Yavuz Kal**: writing – review and editing, resources. **Mehmet Bozkurt Ataman**: writing – review and editing, conceptualization, methodology.

## Ethics Statement

All procedures in this study were compatible with the Animal Experiments Local Ethics Committee guidelines of Bahri Dağdaş International Agricultural Research Institute, Turkey (30.11.2020/114). ARRIVE guidelines were also adhered to by the authors.

## Conflicts of Interest

The authors declare no conflicts of interest.

### Peer Review

The peer review history for this article is available at https://www.webofscience.com/api/gateway/wos/peer‐review/10.1002/vms3.70381.

## Data Availability

The data supporting the findings of this study are available from the corresponding author (bulent.bulbul@deu.edu.tr).
